# Development of a Set of Microsatellite Markers to Investigate Sexually Antagonistic Selection in the Invasive Ant *Nylanderia fulva*

**DOI:** 10.3390/insects12070643

**Published:** 2021-07-15

**Authors:** Pierre-Andre Eyer, Megan N. Moran, Alexander J. Blumenfeld, Edward L. Vargo

**Affiliations:** Department of Entomology, Texas A & M University, 2143 TAMU, College Station, TX 77843-2143, USA; mnm32@exchange.tamu.edu (M.N.M.); alex93@tamu.edu (A.J.B.); ed.vargo@tamu.edu (E.L.V.)

**Keywords:** intralocus sexual conflict, reproductive system, invasive species, haplodiploids, social insects

## Abstract

**Simple Summary:**

The two sexes of a species usually exhibit phenotypic differences, such as in behavior, body size or color. They, however, share most of their genomes, preventing fixation of distinct alleles for genes coding for those traits in each sex. The different optima between the sexes on these loci lead to genomic conflicts, called sexually antagonistic selection (SAS). Under SAS, distinct alleles are therefore selected in each sex. In the invasive tawny crazy ant, *Nylanderia fulva,* a genomic region is under SAS, while the rest of the genome is randomly selected in males and females. Here, we provide a suite of 15 polymorphic microsatellite markers located in the SAS genomic region to study the origin and evolution of SAS in *N. fulva*. These markers have allelic frequencies that are highly different between males and females. All males carry only a subset of the alleles present in the population, while females are reliably heterozygous, with one allele from the male gene pool and a different allele inherited from their mother. The SAS markers may be used to test for the strength and the extent of the genomic regions under SAS in both the native and introduced ranges of *N. fulva*. These markers may serve to answer similar questions in other introduced species of the *Nylanderia* genus, yielding insights into the origin and evolution of SAS within and among species of the genus *Nylanderia*.

**Abstract:**

Sexually antagonistic selection (SAS) occurs when distinct alleles are differentially selected in each sex. In the invasive tawny crazy ant, *Nylanderia fulva,* a genomic region is under SAS, while the rest of the genome is randomly selected in males and females. In this study, we designed a suite of 15 microsatellite markers to study the origin and evolution of SAS in *N. fulva*. These SAS markers were polymorphic, with allelic frequencies that are highly different between males and females. All haploid males carry only a subset of the alleles present in the population, while females are reliably heterozygous, with one allele from the male gene pool and a different allele inherited from their mother. In addition, we identified six polymorphic markers not associated with SAS and six markers yielding consistent, yet monomorphic, amplification in the introduced range of this species. Reaction condition optimizations allowed all retained markers to be co-amplified in four PCR mixes. The SAS markers may be used to test for the strength and the extent of the genomic regions under SAS in both the native and introduced ranges of *N. fulva*, while the set of non-SAS loci may be used to assess the invasion route of this species. Overall, the application of these microsatellite markers will yield insights into the origin and evolution of SAS within and among species of the genus *Nylanderia*.

## 1. Introduction

Within a sexually reproducing species, males and females share a common optimum for most of their traits. However, they may differ in their optima for some specific traits, leading to sexually antagonistic selection (SAS) [1,2]. SAS occurs when specific alleles provide an advantage to one sex while being harmful to the other. Males and females share a common autosomal genome, which usually prevents each sex from independently fixing different alleles [3,4]. SAS was thought to be resolved through the evolution of sex chromosomes, enabling the sexes to overcome the constraint of a shared genome by allowing each sex to separately fix beneficial alleles in distinct sex chromosomes, thus preventing deleterious recombination between them [5,6].

The invasive tawny crazy ant *Nylanderia fulva* represents a unique case of SAS, as it occurs in a sexually reproducing haplodiploid species lacking sex chromosomes [7]. As with many Hymenopteran species, the sex of this ant species is determined by heterozygosity at the complementary sex-determining locus (CSD) [8]. Fertilized diploid eggs develop into females when heterozygous at this locus. In contrast, unfertilized haploid eggs develop into males, as they are hemizygous at this locus. *Nylanderia fulva* is native to South America and has been introduced in the Southern USA [9,10,11]. In the invasive population of this species, a set of alleles is differentially selected in each sex, while the rest of the genome is randomly transmitted [7]. This SAS region (i.e., differentially selected) was estimated to represent ∼3% of the genome based on scaffolds displaying significant values of outbreeding in females, indicating that daughters inherit different alleles from each parent. Twelve microsatellite markers were developed for *N. fulva* before SAS in this species was discovered [9]. Nine of these markers were located in randomly inherited regions, and three were located in SAS regions [7]. Microsatellite analyses at these three SAS loci revealed that daughters (workers and queens) preferentially carry alleles from their mothers, and sons preferentially carry alleles from their grandfathers (males have no fathers). Consequently, females and males exhibit strongly divergent genotypes, with females being nearly 100% heterozygous for markers located in this genomic region [7]. For two of these markers, all males carried a single allele (arbitrarily called allele A), while almost all females were heterozygous, with allele A and another allele (B or C), resulting in either A/B or A/C genotypes. Although these three microsatellite markers allow for the identification of complex DNA inheritance between the sexes, their weak polymorphism (respectively, 2, 3 and 3 alleles for *L02*, *L06* and *L07*) prevents any further analyses. For example, it hampers the determination of whether a) the male gene pool consistently exhibits a single allele, or b) the allelic diversity in the male gene pool is systematically lower than the females’. Similarly, it prevents a robust assessment of whether microsatellite markers located in SAS genomic regions experience a comparable loss of allelic diversity to those located in non-SAS regions after the bottleneck event following this ant’s introduction to the USA. Overall, developing a robust set of microsatellite markers is required to properly answer these questions. Ultimately, these findings may provide insights into the strength of the selective pressures acting upon the male and female gene pools and illuminate the origin and evolution of SAS in this haplodiploid species.

Microsatellite loci are the primary marker of choice to study mating systems, such as the diversity of modes of reproduction and kinship structures observed in ants [12,13,14,15,16,17,18]. These co-dominant markers are usually highly polymorphic, randomly spread throughout the genome and considered as neutral markers [19]. However, in this study, we aimed to design microsatellite markers specifically informative to study SAS in *Nylanderia fulva*. For this purpose, these SAS markers are not randomly spread throughout the genome but instead located in the SAS region(s). They therefore cannot be considered neutral markers, as this genomic region is under strong SAS. Finally, although we sought to find markers with high polymorphism, we did not simply discard markers with no/low polymorphism because such markers could be informative in investigating the selective regimes faced in each sex, as well as the consequence of the bottleneck on this genomic region.

In this study, we designed a set of novel microsatellite loci for *N. fulva*. We tested 29 expected SAS markers located in scaffolds exhibiting significant negative values of *F_IS_* in females. As a positive control, we tested eight expected new SAS markers located in the same three scaffolds containing the previous microsatellites showing an SAS pattern (*L02*, *L06* and *L07*). As a negative control, we also tested six additional expected non-SAS markers located in randomly inherited scaffolds (i.e., scaffolds showing no deviation from the Hardy–Weinberg equilibrium).

## 2. Materials and Methods

### 2.1. Microsatellite Primer Design

The software QDD v. 3.1 [20] was used to discover microsatellite repeat motifs within the draft reference genome of *N. fulva* (RefSeq GCF_005281655.1). We set up a threshold of at least five repetitions, excluded mononucleotide repeats and extracted the 200 bp flanking regions on both sides of the repeats for subsequent primer design. Overall, 218,352 reads containing microsatellite repeat motifs were identified among 2808 scaffolds. Based on Eyer et al. (2019) [7], we examined 16 scaffolds with highly negative values of *F_IS_*, suggesting they exhibit an SAS pattern (*F_IS_ >* −0.7). We also examined six scaffolds with moderate levels of outbreeding (−0.1 < *F_IS_ <* −0.7), and scaffolds containing previously identified SAS markers (*L02*: scaffold 156, *F_IS_* = −0.875; *L06*: scaffold 111, *F_IS_* = −0.488; and *L07:* scaffold 120, *F_IS_* = −0.9). We selected a set of 37 loci (33, 3 and 1 with di-, tri- and tetranucleotide repeats, respectively) among these scaffolds and designed the corresponding primers using the online Primer3 software (http://primer3.ut.ee, accessed on 14 July 2021) [21]. We also selected and designed primers for a set of six loci (4, 1 and 1 with di-, tri- and tetranucleotide repeats, respectively) among five randomly inherited scaffolds (scaffold 20, *F_IS_* = 0.001; scaffold 68, *F_IS_* = 0.008; scaffold 67, *F_IS_* = 0.008; scaffold 7, *F_IS_* = 0.037; and scaffold 53, *F_IS_* = 0.024). For each scaffold, we selected loci with the highest number of repeats to maximize polymorphism [22]. Primers were designed to result in a broad size range of PCR products (120 to 400 bp) in order to facilitate multiplex arrangements. Primer sequences, scaffold information, microsatellite repeat information, PCR conditions and multiplexing arrangements are presented in Table 1.

### 2.2. Genetic Procedures

**Figure 1:**
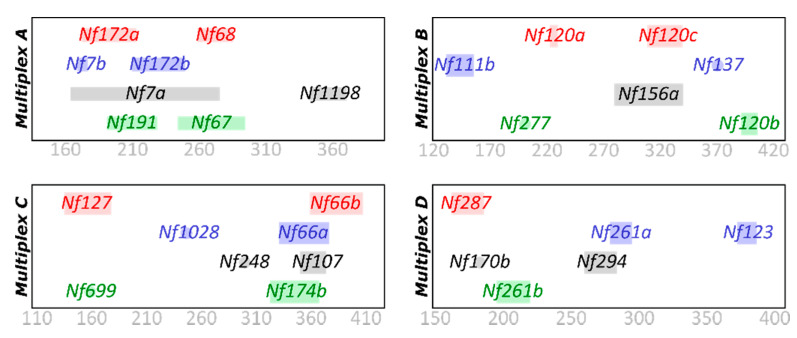
Multiplex arrangement of the four PCR mixes allowing for co-amplification of the 29 microsatellite markers amplifying in this study.

A total of 40 individuals of *N. fulva* were collected in 2017 within its introduced range, including four populations in Texas, and one population each in Mississippi and Louisiana. This sampling included 20 males and 20 females, with females further split into 10 workers and 10 queens. As the invasive range of this species is made of a single vast supercolony [9], the six invasive populations sampled exhibit similar allelic frequencies. For each individual, total genomic DNA was extracted following a modified Gentra Puregene extraction method (Gentra Systems, Inc. Minneapolis, MN, USA). All 43 primer pairs were first tested in standard simplex PCR conditions. Fourteen microsatellite markers were discarded due to inconsistent or nonexistent amplification (Table 1). We further used the M13-tailed primer method to label amplicons to facilitate multiplexing [23]. The M13 tails were attached to the forward primer and 5′-fluorescently labeled with 6-FAM, VIC, PET or NED. Amplicons were amplified using a Bio-Rad thermocycler T100 (Bio-Rad, Pleasanton, CA, USA). PCR products were visualized on an ABI 3500 capillary sequencer and sized against an LIZ500 internal standard (Applied Biosystems, Foster City, CA, USA). Allele scoring was performed using Geneious v.9.1(Biomatters, Auckland, New Zealand) [24]. The 29 microsatellite markers successfully amplifying in simplex conditions were organized into four multiplex groups (A, B, C and D, made of, respectively, 8, 8, 7 and 6 markers) by maximizing the number of loci labeled with the same dye while also avoiding overlaps (Table 1, Figure 1). Although the monomorphic markers (*n* = 6) were conserved in the multiplex arrangement due to their potential polymorphism in the native range of *N. fulva*, they were discarded from further analyses in the present study.

### 2.3. Confirming Inheritance Patterns of SAS and Non-SAS Markers

For each marker, the number of alleles and allelic frequencies were calculated in each sex and caste, as well as for the entire dataset using GENEPOP on the web [25]. This software was also used to compare allelic frequencies between the sexes using a genic test of differentiation. The software FSTAT was used to calculate *F_IS_*, departures from the HWE and the expected (He) and observed (Ho) heterozygosities for the female sex [19]. SAS markers are expected to deviate from the HWE by showing a high level of heterozygosity in females, which results in highly negative values of *F_IS_*. Additionally, SAS markers are expected to exhibit alleles differentially selected in each sex, which should lead to differences in allelic frequencies between males and females, and ultimately distinct alleles in each sex. In contrast, non-SAS markers are not expected to deviate from the HWE (i.e., no evidence of outbreeding or inbreeding), and therefore allelic diversities and allelic frequencies are not expected to differ between the sexes in these markers.

## 3. Results and Discussion

All 29 selected microsatellite markers amplified well and yielded clear and readable products. Twenty-three of them displayed polymorphism, ranging from 3 to 12 alleles per marker (Table 2). They are therefore informative for genetic studies of the tawny crazy ant *Nylanderia fulva*. Since all individuals analyzed in this study originate from the US introduced range, these markers are likely to exhibit higher polymorphism in the native range of this species. A similar outcome can be expected for the six markers found to be monomorphic in this study (Table 1).

**Figure 2:**
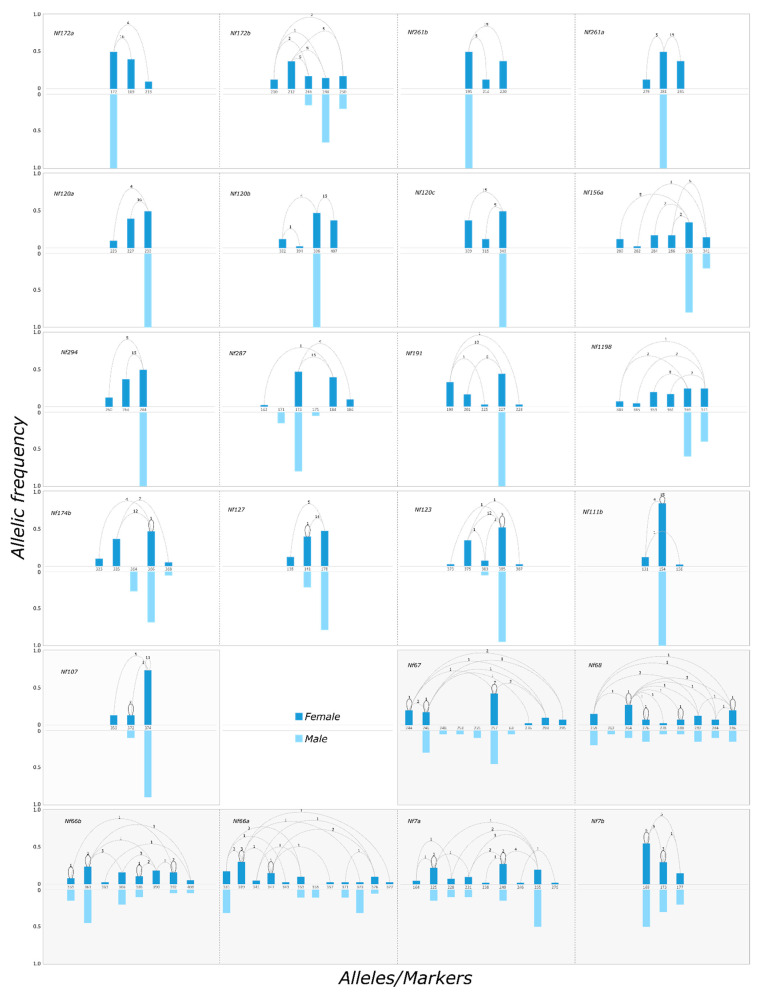
Allelic frequency in the female (dark blue) and male (light blue) gene pools for each polymorphic marker. Dashed lines indicate co-occurring alleles in heterozygous females, while solid lines indicate alleles found in homozygous females. The numbers above the lines indicate the numbers of heterozygous/homozygous individuals. Males are haploid and therefore always hemizygous.

**Figure 3:**
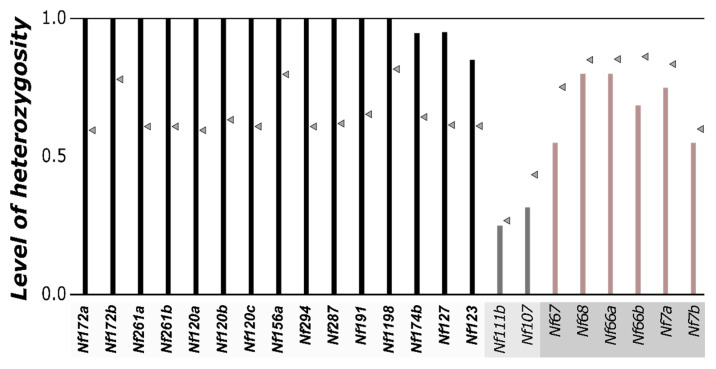
Level of heterozygosity observed in females for each polymorphic marker. Arrows indicate the level of expected heterozygosity.

Fifteen microsatellite markers exhibited a clear deviation from the HWE, with more observed heterozygotes than expected after Bonferroni correction (all *p* < 0.01) (Table 2). Of these markers, 14 exhibited a level of heterozygosity of at least 0.95 (the last one being 0.85; twelve markers were completely heterozygous). Consequently, the *F_IS_* inbreeding coefficient was highly negative in females for these 15 markers (*F_IS_* ± = −0.54 ± 0.16, from −0.23 to −0.71). The allelic frequencies for these markers were also highly different between males and females (all *p* < 0.001). All males consistently carry only a subset of the alleles present in the population (one to three alleles), while females are reliably heterozygous, with one paternal allele and a different allele inherited from their mother (Table 2, Figure 2 and Figure 3). Consequently, allelic frequencies in females never exceed 0.5 (they reach 0.5 when the male gene pool contains a single allele). Of these 15 markers, 7 and 4 markers were located in scaffolds with high and moderate levels of outbreeding, respectively, and 4 markers were located in scaffolds containing identified SAS markers. Overall, these results suggest that these 15 microsatellite markers denote a pattern of SAS in the introduced range of *N. fulva*, with allele numbers for females ranging from three to six (mean ± SD = 4.2 ± 1.22), while allele numbers for males only range from one to three (mean ± SD = 1.66 ± 0.79).

In comparison, six microsatellite markers did not exhibit a deviation from the HWE. In females, these loci displayed similar values of the observed (0.55–0.80) and expected (0.60–0.85) heterozygosity (Figure 3), resulting in a slightly positive inbreeding coefficient (*F_IS_* ± SD = 0.13 ± 0.08; from 0.06 to 0.27). Allele numbers ranged from 3 to 12 at these six loci for the combined male/female dataset (mean ± SD = 8.5 ± 2.75; Table 2), and allelic frequencies were not different between males and females for three out of the six markers (*p* > 0.05). However, for the other three markers, the allelic frequencies were different (*p* < 0.05), most likely stemming from non-sampled alleles among the limited number of samples of each sex (*n* = 20) in regard to the higher number of alleles for these markers (8, 10 and 12). Of these six markers, two were located in one scaffold with moderate levels of outbreeding, and four were located in scaffolds with *F_IS_* inbreeding coefficients close to zero. Overall, these results suggest that these six microsatellite markers are representative of a genomic region within *N. fulva* not under SAS.

Two microsatellite markers showed ambiguous results that prevent us from clearly labeling them as SAS or non-SAS markers. They exhibited low levels of observed heterozygosity in females (0.25 and 0.31) and did not deviate from the HWE. However, allelic frequencies were different between males and females (*p* < 0.05), but *F_IS_* was low to moderate (*F_IS_* = 0.07 and 0.277).

## 4. Conclusions

Overall, we designed a final suite of 29 microsatellite loci yielding consistent amplification for studying SAS in the invasive tawny crazy ant *Nylanderia fulva.* This includes a set of 15 new SAS markers that may be used to study the origin and evolution of SAS in this species. Notably, these markers can be used to decipher whether native populations of *N. fulva* also exhibit genomic regions under SAS, or whether SAS is restricted to the source population that invaded the Southeastern US. Interestingly, different SAS markers show different values of *F_IS_*, with some SAS markers being completely heterozygous in the female castes, while other SAS markers contained a few homozygous females. This result may suggest that, even in the introduced range, the strength of selection is variable across different SAS genomic regions, potentially allowing local recombination events. Similarly, these SAS markers may be used to test for the strength and the extent of the genomic regions under SAS in the native range of *N. fulva* (i.e., whether all 15 markers exhibit a pattern of SAS or only some of them, and whether their levels of heterozygosity are always 100% or lower). This may identify specific genomic regions under different levels of SAS [26,27,28,29,30,31] and provide insights into the strength of sexual antagonism in haplodiploids [32,33,34]. Although we are awaiting confirmation with a larger sampling size, the number of alleles observed in this study seems greater in the non-SAS loci than the SAS loci, which may denote a strong selective pressure associated with SAS. The set of non-SAS microsatellite loci may also be used in conjunction with previously developed markers to assess the genetic diversity, population structure and phylogeography at local and global scales throughout the native and introduced ranges of this species. These findings may provide valuable information regarding the introduction route of *N. fulva* out of South America, inferring the source population(s) and the extent of the bottleneck the invasive US populations have experienced.

The sets of markers developed in this study may also be used for genetic analyses on other species of *Nylanderia*, including the other invasive members of this genus [35]. The genus *Nylanderia* is among the most invasive of all ant genera, with 15 globetrotting species encountered beyond their native ranges [36,37]. Therefore, non-SAS loci may be of use for classical genetic analyses, such as population structure and breeding system analyses for these species. As in *N. fulva*, these markers may also serve to infer the invasion routes of the different introduced species, as well as the series of demographic changes they experienced. Additionally, the SAS loci may be used to investigate if SAS is widespread across this genus, or if it is confined to *N. fulva*. Overall, the application of these microsatellite markers will provide valuable tools for increasing our understanding about the origin and evolution of SAS within and among species of the genus *Nylanderia*.

## Figures and Tables

**Table 1:** Primer sequences, scaffold information, microsatellite repeat information, PCR conditions and multiplexing arrangements for each microsatellite marker tested.

Marker Name	Scaffold	FIS Scaffold	Scaffold FIS Value	MS Motif	# of Repeats	Left	Right	TM	Product Size	Peak Reading *	Color	Multiplex
*Nf172a*	S_172	High	−0.900	CT	33	TACAGCCGCGTTGTTTTCAC	GGCAACATATCAAGAACCCTGT	57	172/213		PET	A
*Nf172b*	S_172	High	−0.900	TC	27	TTAATGAGGGGCCCGTTGAT	GCATGTATGAAAGAGCAGCGA	57	210/250		FAM	A
*Nf137*	S_137	High	−0.900	TA	24	ACGTGTGTTGTGTGTGTGTG	TGGTGCTTTTAATACAGTGGCT	57	370	monomorphic	FAM	B
*Nf1028*	S_1028	High	−0.900	TA	18	GCAATGCCACTCAAGGTCAA	GCTCTGTTGGCCGATTTAAAA	57	249	monomorphic	FAM	C
*Nf699*	S_699	High	−0.900	TA	27	AGTCAATTAAAACGAGTCCTGGA	TGTGTGAAAATACGTGTGTCTAC	57	162	monomorphic	VIC	C
*Nf652*	S_652	High	−0.900	TCT	17	CGGAGATACAAGCGGTCAAA	AGGGAGGTGTGAGTGAAAGG		/	no amplification		
*Nf1198*	S_1198	High	−0.878	CATA	12	GAGACCACATACACCAAAAGGT	GCAGAAAATTAGTTGCGCGA	57	343/371		NED	A
*Nf191*	S_191	High	−0.742	AT	24	TGCGCGTTAATATCTCAAACTCT	TGGATGAAATGAGAGATGTGGG	57	191/229		VIC	A
*Nf248*	S_248	High	−0.764	AG	28	CTATGCACGCTCCTCACTCT	ACCGAGACCTTGTACACACT		295	monomorphic	NED	C
*Nf401a*	S_401	High	−0.667	TA	21	ACGTGTGCATGTTGAGAGAG	TGCCCCTTTCGAAACGTAGA	57	[315]	inconsistent		
*Nf401b*	S_401	High	−0.667	TA	21	CATACCTGCAGCATCCCCTA	TAAGATGCATGCACACACGC	57	[222]	inconsistent		
*Nf170a*	S_170	High	−0.609	GAC	52	CCACAGATCTCGTTCCGTCT	TGAAGGTGCTGAGGAGGATG		/	no amplification		
*Nf170b*	S_170	High	−0.609	AT	24	CGAGTGTCTTTATTTCGCGC	GTCCCAGAAATGAACACCGC	60	172	monomorphic	NED	D
*Nf294*	S_294	High	−0.900	AT	26	GTTTGACGACATTTCTCTGTTCA	CGCAAGTGTAAACGCAATCT	57	260/284		NED	D
*Nf502*	S_502	High	−0.900	TC	30	GGTGGATGAGGGAGTTGGAA	TACCTTCCGCACATAACCCA		[336/367]	inconsistent		
*Nf174a*	S_174	High	−0.889	TC	63	CCCGCTTCGAACATGACAAT	TCATGGAATATCGCATTGTCGT		/	no amplification		
*Nf174b*	S_174	High	−0.889	TA	30	AGCTAACTGACTGACTGCGT	CGATATTCTGCTCGTCGTCAC	57	323/368		VIC	C
*Nf287*	S_287	High	−0.900	AG	30	CGAATTTTATGCTCGCCGGA	GATTTGATTCCAGAGCGCGA	57	162/186		PET	D
*Nf277*	S_277	High	−0.900	CT	34	GCGAGAGAGACGGTATCAC	AGAATTCGATGTACACCGGGT	57	188	monomorphic	VIC	B
*Nf167a*	S_167	High	−0.629	TA	39	AGCAGAGAGAAAGAAATGAGAGT	TTGTTAGGGATAGATCGGAGGA		/	no amplification		
*Nf167b*	S_167	High	−0.629	TC	28	GGGGCTAACTTCATACAGGC	ACCTTCTGCGAATGGTAGCT	57	[281]	inconsistent		
*Nf107*	S_107	Mod.	−0.125	AG	31	ACAAGTCACTTCCGCTGAAAC	CGCAAGGATCAGGTACCGAT	60	350/374		NED	C
*Nf261a*	S_261	Mod.	−0.178	CT	32	ATGCTCTTTGTCACGAGGGA	CGAGAGAAAGGGAAGGGTGA	60	279/295		FAM	D
*Nf261b*	S_261	Mod.	−0.178	TCG	21	CATACTATCTGGGCGGGTGT	CACTGAGAAGATCGCGAGTG	57	195/220		VIC	D
*Nf66a*	S_66	Mod.	−0.439	TA	32	AAACTACGCTCGCAATGCAA	ATGAGAGGGTGTGGAAGAGC	57	331/377		FAM	C
*Nf66b*	S_66	Mod.	−0.439	TA	31	GTGCTCCACTCCAATAATGCT	TGGTCAGGAGTCACGGTAAA	55	359/408		PET	C
*Nf127*	S_127	Mod.	−0.335	AG	31	GCGGCTCGTTAGTGATTCTC	GAGACTCCATTTTGACGGCG	57	135/178		PET	C
*Nf123*	S_123	Mod.	−0.279	AG	28	TGAAAATGTACGCGCGACTT	CCAGCCTTTCAGTGATTCGA	57	373/387		FAM	D
*Nf57*	S_57	Mod.	−0.540	TA	31	GTGTGGAGGAGCAATTTGGG	AATAACGCACTGTCATCCGC	57	[208]	inconsistent		
*Nf120a*	S_120	SAS	−0.900	GA	28	AGAAGCCGCCATCAAGAAGA	GGGAAGATGAGCGCGATCA	60	223/230		PET	B
*Nf120b*	S_120	SAS	−0.900	TC	26	GCCTCTTTATTCGCGGAAGG	AGATTTTACAGCTACGCCGC	57	392/407		VIC	B
*Nf120c*	S_120	SAS	−0.900	AC	24	AGATTGACATTTTCCGCTCTTCA	CCCCATTTGTTCGCTCGTAG	57	309/340		PET	B
*Nf111a*	S_111	SAS	−0.488	GA	32	GGACAAGTTGGAACGGGATG	AACAGAGGAGAACGCGGTAA	57	[308]	inconsistent		
*Nf111b*	S_111	SAS	−0.488	CA	25	GCGTGGATGCTCTTTTCACA	GAAAGTATCTTCTTGCGTgcg	57	131/156		FAM	B
*Nf111c*	S_111	SAS	−0.488	TC	24	CACGCTAAACTGTCATCCGA	CGTGTTGAAGGGAGGGAAGA	62	[162/170]	inconsistent		
*Nf156a*	S_156	SAS	−0.875	AT	43	ACACACTGTACTACTGCGGT	GCGAAATGAGAACGGTAGGT	55	280/341		NED	B
*Nf156b*	S_156	SAS	−0.875	TA	23	CGTAACCTTCGAAATGGCTGT	GTTGCAGAGATCCGAACGAT		/	no amplification		
*Nf20*	S_20	Low	0.000	AG	73	ACTCCTAACTGCTGCCTAATTT	GCACGAATTTACAATTGCGCA		/	no amplification		
*Nf68*	S_68	Low	0.007	GA	32	TACCCACGCATAATCCACCC	CCTCCTTGTTTGTAACGGAAGA	57	259/286		PET	A
*Nf67*	S_67	Low	0.008	TC	36	AAATCCCTGTTTTAAACTGCTGT	AGAACGTTCGAGTGTAGATAGGT	57	244/295		VIC	A
*Nf7a*	S_7	Low	0.038	CAT	36	ACGTGGTTGTTGGTGCATAC	AGCAAGAGAGAGACCGATGT	55	164/276		NED	A
*Nf7b*	S_7	Low	0.038	GGTA	21	TTAGTGGTGCAAAAGGGAAGA	GTTGTGGTGGCAAAGGGTG	60	169/177		FAM	A
*Nf53*	S_53	Low	0.024	TA	33	CCTTGCATCATGTGTGGACC	TCACACGAGGAGACAAGAGG		/	no amplification		

* *Inconsistent peak reading mostly denotes markers with high stutter and similar sizes of alleles, therefore hampering a confident reading of the distinct alleles (especially at heterozygous state).*

**Table 2:** Number of alleles for each sex and caste, level of observed and expected heterozygosity and *F_IS_* for each polymorphic microsatellite marker. We also indicate whether or not each marker follows a pattern of SAS.

Marker Name	Number of Alleles				Heterozygosity		HW Sign.	FIS	M-F Diff.	Genomic Region
	Overall	Workers	Queens	Males	Obs.	Exp.				
*Nf172a*	3	3	3	1	1.00	0.59	***	−0.712	***	*SAS*
*Nf172b*	5	5	5	3	1.00	0.78	**	−0.293	***	*SAS*
*Nf1198*	6	6	6	2	1.00	0.82	**	−0.232	***	*SAS*
*Nf191*	5	4	4	1	1.00	0.68	***	−0.489	***	*SAS*
*Nf294*	3	3	3	1	1.00	0.61	***	−0.670	***	*SAS*
*Nf174b*	5	4	3	3	0.95	0.64	***	−0.493	***	*SAS*
*Nf287*	6	3	4	3	1.00	0.62	***	−0.642	***	*SAS*
*Nf120a*	3	3	3	1	1.00	0.59	***	−0.712	***	*SAS*
*Nf120b*	4	4	3	1	1.00	0.63	***	−0.603	***	*SAS*
*Nf120c*	3	3	3	1	1.00	0.61	***	−0.670	***	*SAS*
*Nf156a*	6	5	6	2	1.00	0.80	**	−0.263	***	*SAS*
*Nf261a*	3	3	3	1	1.00	0.61	***	−0.670	***	*SAS*
*Nf261b*	3	3	3	1	1.00	0.61	***	−0.670	***	*SAS*
*Nf127*	3	3	3	2	0.95	0.61	**	−0.570	***	*SAS*
*Nf123*	5	4	3	2	0.85	0.61	**	−0.407	***	*SAS*
*Nf66a*	12	7	9	6	0.80	0.85	*NS*	0.063	***	*non-SAS*
*Nf66b*	8	7	7	6	0.68	0.86	*NS*	0.211	*	*non-SAS*
*Nf68*	9	6	8	9	0.80	0.85	*NS*	0.060	*NS*	*non-SAS*
*Nf67*	10	6	5	6	0.55	0.75	*NS*	0.273	***	*non-SAS*
*Nf7a*	9	7	7	5	0.75	0.83	*NS*	0.104	*NS*	*non-SAS*
*Nf7b*	3	3	3	3	0.55	0.60	*NS*	0.085	*NS*	*non-SAS*
*Nf111b*	3	3	2	1	0.25	0.27	*NS*	0.069	*	*unclear*
*Nf107*	3	3	3	2	0.32	0.43	*NS*	0.278	*	*unclear*

* *p* < 0.05, ** *p* < 0.01, and *** *p* < 0.001.

## Data Availability

The data supporting the reported results have been deposited in the Open Science Framework data repository at doi:10.17605/OSF.IO/GH7KS.
